# Interleukin-6 in hemolytic anemias: from inflammatory pathways to therapeutic targeting

**DOI:** 10.3389/fimmu.2026.1919619

**Published:** 2026-08-18

**Authors:** Manjun Zhao, Xin Yu

**Affiliations:** Department of Internal Medicine, Peking Union Medical College Hospital, Chinese Academy of Medical Sciences and Peking Union Medical College, Beijing, China

**Keywords:** autoimmune hemolytic anemia, hemolytic anemia, hepcidin, inflammation, interleukin-6, JAK/STAT, siltuximab, tocilizumab

## Abstract

Hemolytic anemias comprise inherited and acquired disorders characterized by premature red blood cell destruction. Increasing evidence indicates that many forms of hemolysis are accompanied by sustained inflammatory and immune activation. Red blood cell breakdown releases damage-associated molecular patterns, including free heme and hemoglobin, which activate innate immune pathways, endothelial cells and coagulation cascades. Interleukin-6 (IL-6) is a central mediator linking hemolysis to systemic inflammation, iron restriction and autoimmunity. In this review, we use a heuristic dual-axis framework in which hemolysis-induced IL-6 promotes hepcidin-dependent iron sequestration and acute-phase responses, while also supporting Tfh and Th17 responses, impairing regulatory T-cell function and sustaining autoreactive or alloreactive B cells. The conceptual advance of this framework is not to propose IL-6 as a uniform pathogenic mechanism, but to position IL-6 differently across hemolytic disorders according to the dominant initiating biology and level of evidence. These pathways may aggravate anemia, thrombosis and organ injury. We summarize evidence for IL-6 involvement in autoimmune hemolytic anemia, Castleman disease-associated cytopenias, sickle cell disease, thalassemia, paroxysmal nocturnal hemoglobinuria, congenital red-cell membrane and enzyme defects, and hyperhemolysis syndrome. We also discuss therapeutic strategies targeting IL-6, IL-6 receptor, JAK/STAT signaling and IL-6 trans-signaling. Current clinical evidence remains limited, but IL-6-directed therapy may be rational in selected refractory immune-mediated or macrophage-activation-associated hemolytic disorders. Future studies should prioritize mechanism-based biomarkers and prospective, biomarker-stratified trials.

## Highlights

- Hemolysis releases DAMPs that activate IL-6-related inflammatory pathways.
- IL-6 links hemolysis to hepcidin induction, iron restriction and anemia of inflammation.
- IL-6 promotes Tfh/Th17 responses and sustains autoreactive or alloreactive B cells.
- IL-6 blockade may be useful in selected refractory immune-mediated or macrophage-activation-associated hemolytic disorders.
- Biomarker-guided trials are needed before routine IL-6-targeted therapy can be recommended.

## 1 Introduction

Hemolytic anemias (HA) are defined by shortened red blood cell (RBC) survival caused by intrinsic erythrocyte defects or extrinsic factors in the circulation. It includes acquired immune-mediated disorders such as autoimmune hemolytic anemia (AIHA), as well as inherited hemoglobinopathies including sickle cell disease and thalassemia (1–4). Although traditionally viewed as “purely hematologic” conditions, many hemolytic disorders are now recognized as inflammatory and immune-mediated diseases.

Hemolysis provides continuous inflammatory stimulation. RBC destruction releases damage-associated molecular patterns (DAMPs), including free heme, hemoglobin and ATP. These molecules activate Toll-like receptors, inflammasome pathways and endothelial cells, promoting cytokine release, coagulation activation and vascular dysfunction (5–7). In parallel, some forms of HA, particularly AIHA, Evans syndrome and Castleman disease-associated cytopenias, are directly driven by immune dysregulation, loss of tolerance and autoantibody formation (1, 2).

Interleukin-6 (IL-6) connects these processes. It is produced by monocytes, macrophages, lymphocytes, fibroblasts, endothelial cells and stromal cells in response to infection, inflammation and tissue injury. IL-6 drives acute-phase responses, induces hepcidin, modulates iron metabolism and shapes adaptive immunity. Elevated IL-6 is a feature of many autoimmune and autoinflammatory disorders and has been associated with disease activity, organ injury and treatment resistance (8–10).

**Figure 1:**
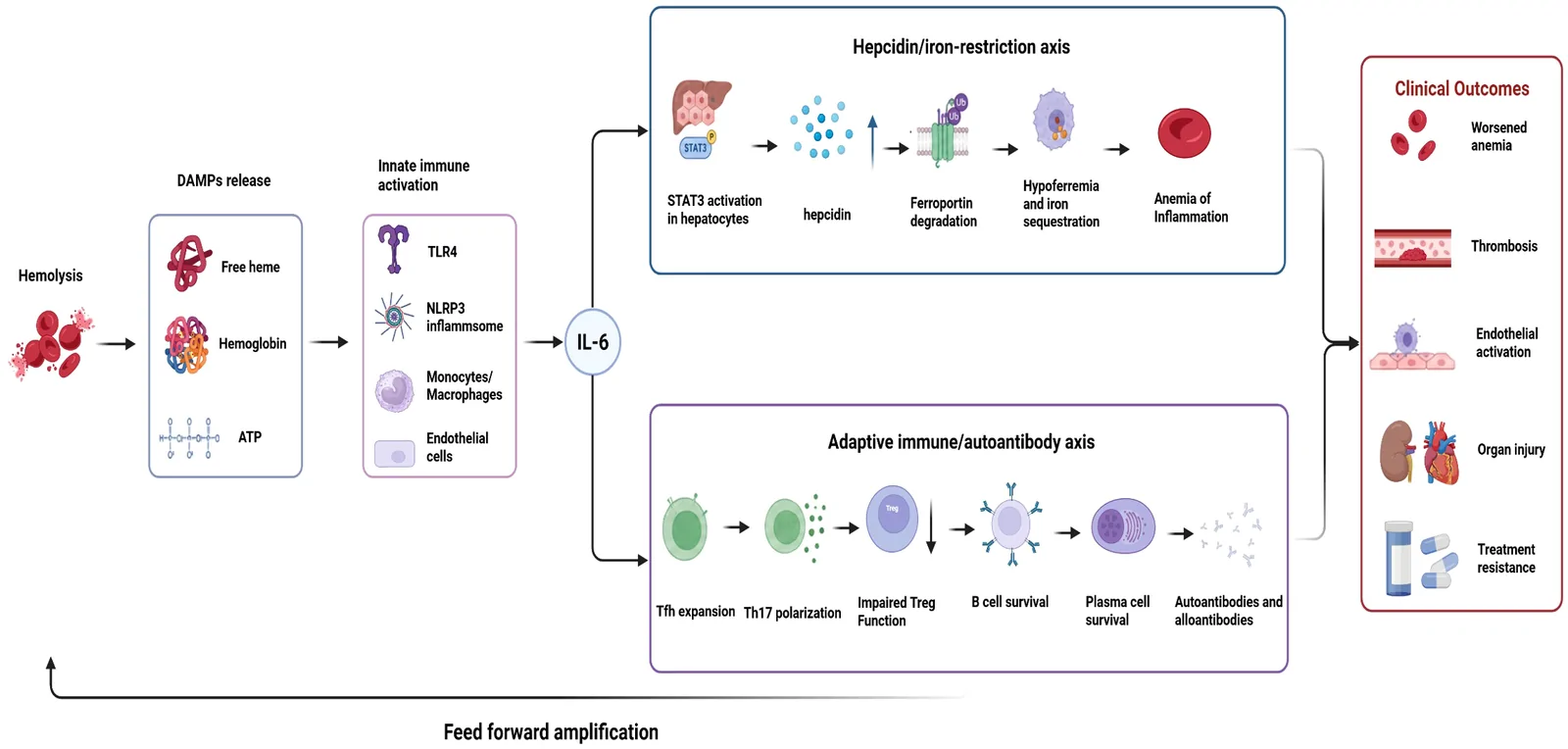
Proposed model linking hemolysis to IL-6-driven pathology. Hemolysis-derived DAMPs induce IL-6 production by innate immune and stromal cells. IL-6 then amplifies hepcidin-mediated iron restriction and adaptive immune activation, thereby worsening anemia, thrombosis and organ injury.

We propose a heuristic hemolysis-DAMPs-IL-6 framework with two downstream axes. First, IL-6 activates a hepcidin-centered iron-restriction and acute-phase pathway, adding anemia of inflammation to primary hemolysis. Second, IL-6 promotes an adaptive immune pathway by supporting T follicular helper (Tfh) and Th17 responses, limiting regulatory T-cell function and sustaining autoreactive or alloreactive B cells. Together, these pathways can worsen anemia, thrombosis and tissue injury (**Figure 1**). Compared with previous reviews that have mainly summarized IL-6 biology or individual hemolytic diseases, the intended novelty of this review is the disease-specific positioning of IL-6 as a potential primary driver, immune amplifier, thrombo-inflammatory modifier, or acute macrophage-activation mediator. This classification is proposed as a conceptual hypothesis rather than a validated taxonomy.

This review summarizes IL-6 biology relevant to hemolysis, discusses its role across major hemolytic disorders, and evaluates therapeutic strategies targeting IL-6, IL-6 receptor, JAK/STAT signaling and IL-6 trans-signaling. Particular attention is given to refractory immune-mediated hemolysis and hyperhemolysis syndrome, where IL-6-directed therapy has begun to emerge as a rational but still experimental approach.

### 1.1 Literature search strategy

This narrative review was based on searches of PubMed, Web of Science and Google Scholar for articles published up to June 1, 2026. Search terms included combinations of “interleukin-6”, “IL-6”, “hemolysis”, “hemolytic anemia”, “autoimmune hemolytic anemia”, “Evans syndrome”, “Castleman disease”, “sickle cell disease”, “thalassemia”, “paroxysmal nocturnal hemoglobinuria”, “hyperhemolysis syndrome”, “hepcidin”, “DAMPs”, “heme”, “tocilizumab”, “siltuximab”, “JAK inhibitor” and “trans-signaling”. Priority was given to mechanistic studies, clinical cohorts, clinical trials, consensus statements, guidelines, and case reports or case series describing IL-6-directed therapy in hemolytic or immune cytopenic contexts. Additional references were identified from the bibliographies of relevant articles. Because this was a narrative review, no meta-analysis or certainty grading was performed. However, throughout the manuscript we distinguish mechanistic plausibility, observational associations, case-based therapeutic signals and higher-level clinical evidence when interpreting the role of IL-6.

## 2 IL-6 biology relevant to hemolysis and autoimmunity

### 2.1 IL-6 signaling modalities

**Figure 2:**
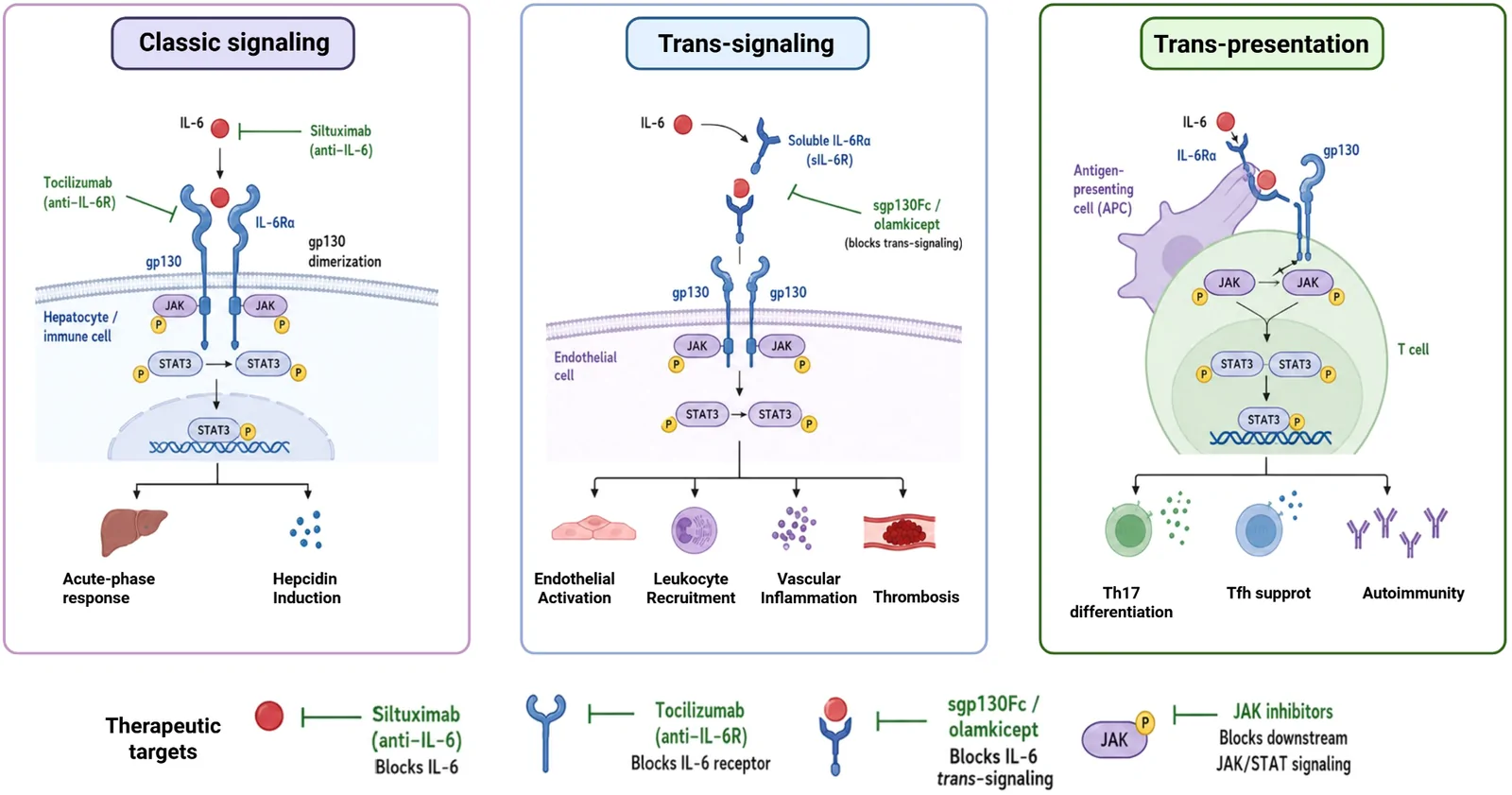
IL-6 signaling modalities and pharmacological target points. IL-6 acts through classic signaling, trans-signaling and trans-presentation, with different implications for hepatic acute-phase responses, vascular inflammation and adaptive immune activation.

IL-6 signals through three main mechanisms (**Figure 2**): classic signaling, trans-signaling and trans-presentation. In classic signaling, IL-6 binds to membrane-bound IL-6 receptor (mIL-6R), expressed mainly on hepatocytes, neutrophils, monocytes and selected T-cell subsets. The IL-6–mIL-6R complex associates with gp130, leading to Janus kinase activation and phosphorylation of STAT proteins, especially STAT3, as well as activation of MAPK and PI3K pathways (11–13).

In trans-signaling, IL-6 binds soluble IL-6 receptor (sIL-6R), generated by alternative splicing or proteolytic shedding. The IL-6-sIL-6R complex activates gp130 on cells that lack mIL-6R, including endothelial and stromal cells. This expands the range of IL-6-responsive tissues and is considered particularly important in chronic inflammation, vascular activation and organ damage (11–13).

Trans-presentation occurs when IL-6-IL-6R complexes on antigen-presenting cells activate gp130 on T cells through direct cell-cell interaction. This pathway contributes to Th17 differentiation and autoimmune inflammation. In hemolytic disorders, endothelial activation and monocyte stimulation may favor trans-signaling, whereas lymphoid interactions involving dendritic cells and autoreactive T cells may involve trans-presentation. These distinctions provide a rationale for selectively targeting IL-6 trans-signaling in vascular complications while preserving some homeostatic functions of classic IL-6 signaling (14).

Upstream of IL-6, hemolysis-derived DAMPs can activate an IL-1β-IL-6 cascade. Free heme and hemoglobin stimulate TLR4-related innate immune signaling, whereas extracellular ATP and heme-associated stress can promote NLRP3 inflammasome activation and IL-1β maturation. IL-1β then induces IL-6 production by monocytes, macrophages and endothelial cells, thereby linking intravascular or extravascular hemolysis to both innate inflammation and the adaptive immune pathways discussed below (5–7).

Macrophages and endothelial cells are particularly relevant sources and targets of IL-6 in hemolytic diseases. During erythrophagocytosis, macrophages process heme through heme oxygenase pathways, increase ferritin expression to store liberated iron, and may produce IL-6 when heme burden or inflammatory co-signals exceed homeostatic capacity. IL-6 in turn promotes hepatic hepcidin production and favors macrophage iron retention, contributing to functional iron restriction. Endothelial cells respond especially to IL-6 trans-signaling by increasing adhesion molecules, chemokines and leukocyte recruitment, thereby amplifying vascular inflammation in disorders such as sickle cell disease and paroxysmal nocturnal hemoglobinuria (11–17).

### 2.2 IL-6, hepcidin and iron metabolism

A major way in which IL-6 contributes to anemia is through hepcidin regulation. Hepcidin, produced by hepatocytes, is the key hormone controlling systemic iron homeostasis. It binds ferroportin on enterocytes, macrophages and hepatocytes, causing ferroportin internalization and degradation. This reduces intestinal iron absorption and iron release from stores (15–17).

IL-6 is the dominant inflammatory inducer of hepcidin transcription through JAK/STAT3 signaling in hepatocytes. During infection or inflammation, IL-6-driven hepcidin upregulation causes hypoferremia and iron sequestration in the reticulo-endothelial system, limiting iron availability for erythropoiesis and contributing to anemia of inflammation (15, 16). IL-6 also reduces transferrin and increases ferritin, further shifting iron toward storage rather than erythroid use. At the cellular level, this pathway is reinforced by erythrophagocytosis-associated heme uptake in macrophages, which increases intracellular iron handling and ferritin expression while IL-6-hepcidin signaling limits iron export through ferroportin.

In chronic hemolytic disorders such as sickle cell disease and thalassemia, this pathway intersects with signals from ineffective erythropoiesis, including growth differentiation factor-15 and erythroferrone, which suppress hepcidin. Hepcidin may therefore be low relative to total iron burden but still sufficient to impair iron mobilization for erythropoiesis. This creates the paradoxical coexistence of systemic iron overload and functional iron restriction. In this setting, IL-6 is often secondary to hemolysis and tissue injury but becomes a proximal driver of iron-restricted erythropoiesis.

### 2.3 IL-6 and adaptive immunity

IL-6 is also a central regulator of adaptive immunity. It promotes B-cell proliferation, survival and differentiation into antibody-secreting plasma cells. Together with IL-21, IL-6 supports Tfh-cell development and function, thereby enhancing germinal-center reactions and antibody production, including autoantibody formation (18).

In T cells, IL-6 cooperates with TGF-β to promote differentiation of naïve CD4-positive T cells into Th17 cells while inhibiting the generation and stability of Foxp3^+^ regulatory T cells. This Th17/Treg imbalance is a common feature of autoimmune disease and contributes to persistent inflammation and loss of tolerance (19, 20). IL-6 also sustains effector and memory T-cell responses and enhances cytokine production.

These effects are particularly relevant to AIHA and other immune cytopenias. In idiopathic warm AIHA and Castleman disease-associated cytopenias, IL-6 often appears to act upstream of disease flares and treatment resistance. In hereditary hemolytic anemias, IL-6 is more commonly induced downstream of hemolysis but may still amplify alloimmune or autoimmune responses.

### 2.4 Conceptual framework: disease-specific positioning of IL-6

Across hemolytic syndromes, IL-6 can be conceptualized through two partly over-lapping axes. The first is a hepcidin/iron-maldistribution axis, in which IL-6–STAT3 signaling induces hepcidin, hypoferremia and iron sequestration, thereby adding an anemia-of-inflammation component to primary hemolysis. The second is an adaptive immune/autoantibody axis, in which IL-6 sustains Tfh and Th17 responses, impairs regulatory T-cell activity and promotes the survival of autoreactive or alloreactive B cells (**Figure 1**).

**Figure 3:**
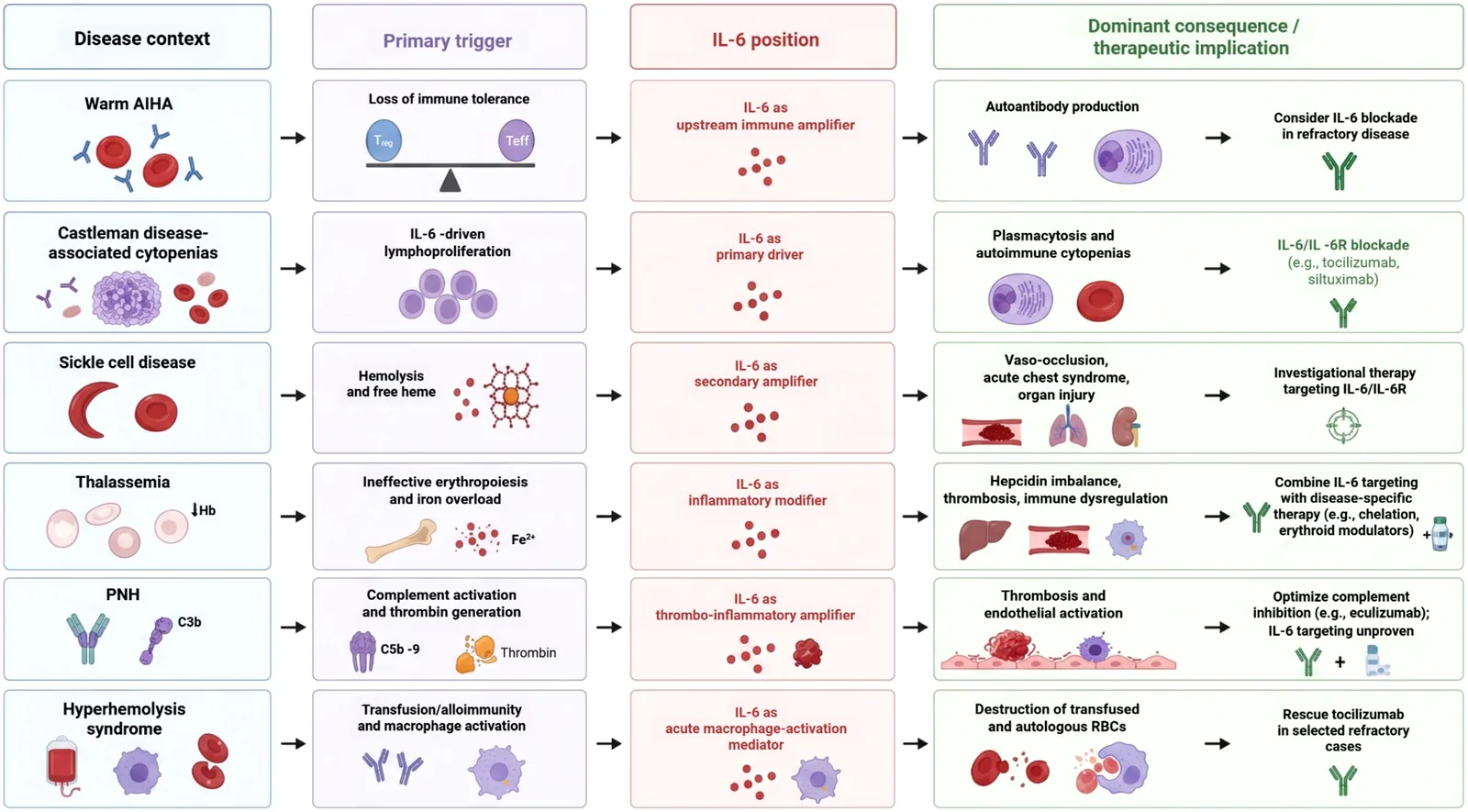
Disease-specific positioning of IL-6 in hemolytic anemias. IL-6 may act as a primary immune driver, secondary inflammatory amplifier or an acute mediator of macrophage activation depending on the hemolytic context.

A central premise of this review is that IL-6 should not be assigned a uniform pathogenic role across all hemolytic anemias, and that its classification requires explicit criteria. We positioned IL-6 according to four considerations: the dominant initiating trigger of hemolysis, the temporal relationship between IL-6 elevation and disease activity, the prevailing biological signature involved-hepcidin/iron restriction, adaptive autoimmunity, endothelial-coagulation activation or macrophage activation and the strength of therapeutic evidence supporting IL-6-directed intervention. Instead, its position varies according to the initiating disease biology. In Castleman disease-associated AIHA or Evans syndrome, IL-6 may function as a primary driver of systemic inflammation, plasmacytosis, hypergammaglobulinemia and autoantibody-mediated cytopenias. In primary warm AIHA, IL-6 is more plausibly an upstream immune amplifier that supports autoreactive B-cell responses, Tfh-cell activity, Th17 polarization and impaired regulatory immune control. In sickle cell disease, thalassemia and paroxysmal nocturnal hemoglobinuria, IL-6 is better viewed as a secondary amplifier of hemolysis-driven inflammation, endothelial activation, coagulation and iron dysregulation. In hyperhemolysis syndrome, IL-6 may contribute to an acute macrophage-activation phenotype that promotes destruction of both transfused and autologous erythrocytes. This disease-specific positioning should therefore be interpreted as a conceptual hypothesis rather than a validated taxonomy, and it requires prospective biomarker-based validation. This disease-specific positioning is essential for interpreting therapeutic reports and for designing future biomarker-stratified trials (**Figure 3**).

## 3 IL-6 in autoimmune and Castleman disease-associated hemolysis

### 3.1 Warm autoimmune hemolytic anemia

Warm autoimmune hemolytic anemia (wAIHA) is the most common form of AIHA. It is characterized by IgG autoantibodies that bind RBC antigens at 37 °C, leading mainly to extravascular hemolysis in the spleen and liver (1, 2). Although B-cell dysregulation and autoantibody production are central, increasing evidence suggests that IL-6 contributes to disease activity and treatment resistance.

Several studies have reported higher plasma IL-6 levels in active wAIHA than in healthy controls. IL-6 concentrations correlate with hemolytic markers, including hemoglobin, lactate dehydrogenase and bilirubin. In some cohorts, IL-6 levels decline during remission. Higher baseline IL-6 has also been associated with steroid-refractory disease and shorter steroid-free remission, suggesting potential value as a biomarker of disease activity and treatment response (21, 22) However, current data remain limited to small observational studies.

Mechanistically, IL-6 can promote loss of B-cell tolerance and expansion of auto-reactive clones. It directly supports B-cell survival and plasma-cell differentiation and indirectly enhances humoral immunity through Tfh cells and IL-21. It also promotes Th17 differentiation while limiting regulatory T-cell development, creating a cytokine environment favorable to chronic autoimmunity. *In vitro* studies using B cells from wAIHA patients indicate that IL-6 stimulation increases activation markers and immunoglobulin production (21).

Emerging evidence also suggests a link between IL-6 and epigenetic regulation in wAIHA. Altered DNA methylation profiles in peripheral B cells have been associated with disease progression, and IL-6 may influence methylation of genes involved in B-cell activation and survival (23). These mechanisms could reinforce autoreactive B-cell phenotypes and persistent autoantibody production (24). Overall, IL-6 appears to be an important node in the B- and T-cell crosstalk underlying wAIHA, although evidence in primary wAIHA remains insufficient.

### 3.2 Castleman disease-associated AIHA and Evans syndrome

Multicentric Castleman disease (MCD), particularly idiopathic MCD, is a prototypical IL-6-driven lymphoproliferative disorder characterized by generalized lymph-adenopathy, systemic inflammation, polyclonal plasmacytosis and autoimmune manifestations (25, 26). AIHA, immune thrombocytopenia and Evans syndrome are clinically important complications.

Lymph node tissue from idiopathic MCD shows marked IL-6 overexpression, and circulating IL-6 levels correlate with constitutional symptoms, acute-phase reactants and disease activity. IL-6 promotes B-cell survival, plasma-cell differentiation, hypergammaglobulinemia and autoantibody production against RBCs and platelets. It also supports Th2 and Th17 polarization, further amplifying humoral autoimmunity and tissue inflammation (25–27).

Experimental models support a causal role for IL-6 in Castleman-like pathology. IL-6-overexpressing mice develop lymphadenopathy, splenomegaly, plasmacytosis and anemia resembling human Castleman disease. Clinically, IL-6 pathway blockade has transformed the management of idiopathic MCD. Tocilizumab, which blocks IL-6 receptor, and siltuximab, which neutralizes IL-6, improve systemic symptoms, lymphadenopathy and inflammatory markers (25, 27).

IL-6-targeted therapy has also shown benefit in MCD-associated AIHA and Evans syndrome. Case reports describe severe AIHA refractory to high-dose corticosteroids and rituximab that responded rapidly to tocilizumab, sometimes without additional immunosuppression (28, 29). Siltuximab has induced durable hematologic responses in mixed AIHA and Evans syndrome associated with MCD after failure of multiple therapies (30, 31).

These observations support IL-6 as a mechanistic link between lymphoid hyperplasia and autoimmune blood-cell destruction in Castleman disease. Nevertheless, the evidence base consists mainly of uncontrolled case reports, small series and registry data. Not all patients respond, highlighting the need for predictive biomarkers such as baseline IL-6, C-reactive protein and plasmablast signatures.

## 4 IL-6 in non-autoimmune, complement-mediated and transfusion-associated hemolysis

### 4.1 Sickle cell disease

Sickle cell disease (SCD) is characterized by chronic hemolysis, vaso-occlusive crises, ischemia-reperfusion injury and sustained inflammation (3, 32). Even at steady state, patients with SCD show elevated pro-inflammatory cytokines, including IL-6, reflecting ongoing hemolysis and tissue injury (33).

IL-6 levels are associated with clinical severity. During vaso-occlusive crises, serum IL-6 is higher than at steady state and may correlate with episode duration. In acute chest syndrome, IL-6 concentrations in sputum and bronchoalveolar lavage fluid can be markedly higher than in plasma, indicating intense local pulmonary inflammation (34, 35). High IL-6 has also been linked to chronic complications such as leg ulcers.

Mechanistically, intravascular hemolysis releases free heme and hemoglobin, which activate TLR4 and other pattern-recognition receptors on monocytes, macrophages and endothelial cells (6, 7). Free heme, extracellular ATP and ischemia-reperfusion injury may also activate inflammasome-related pathways, leading to IL-1β production that further stimulates IL-6 release. This induces IL-6 production and endothelial activation. IL-6 then promotes adhesion molecule and chemokine expression, leukocyte recruitment and vascular inflammation, contributing to RBC and leukocyte adhesion, microvascular occlusion and organ ischemia. Through trans-signaling, IL-6 may act on endothelial and stromal cells that express gp130 but little membrane IL-6 receptor, thereby expanding vascular inflammatory responses beyond classical IL-6-responsive cells.

IL-6 may also contribute to immune dysregulation and organ injury in SCD. Persistent IL-6 elevation can enhance production of IL-1β, IL-8 and TNF-α, promote B-cell activation and support autoantibody formation. In SCD mouse models, IL-6 expression is increased in the heart and lung, and heme exposure further increases cardiac IL-6 and profibrotic gene expression (36). IL-6-related signaling has also been implicated in chronic inflammation and multi-tissue injury in experimental SCD (37).

Although IL-6 blockade has not been systematically studied in SCD, mechanistic and correlative data support exploration in selected complications, such as acute chest syndrome or hyperhemolysis syndrome. Case-based experience with tocilizumab in acute chest syndrome suggests possible benefit but remains insufficient to support routine use.

### 4.2 Thalassemia

Thalassemia is caused by impaired globin synthesis and is characterized by ineffective erythropoiesis, chronic hemolysis, anemia and iron overload (4). Many patients also show evidence of chronic inflammation, with elevated IL-6 reported in β-thalassemia compared with healthy controls (38, 39).

The sources of IL-6 in thalassemia are multifactorial. Ineffective erythropoiesis and intramedullary hemolysis release DAMPs that activate innate immune cells. Repeated transfusions and iron-mediated tissue injury further stimulate mononuclear phagocytes. IL-6 may worsen anemia by suppressing erythroid progenitor proliferation, reducing erythropoietin production and limiting iron availability for erythropoiesis (40).

IL-6 is particularly relevant to iron metabolism. Ineffective erythropoiesis suppresses hepcidin through erythroferrone and related signals, promoting intestinal iron absorption and iron overload. In contrast, inflammation-induced IL-6 increases hepcidin. The resulting hepcidin level reflects the balance between erythroid suppression and inflammatory induction. Although hepcidin is often inappropriately low relative to iron burden, IL-6 may still promote iron retention in macrophages and hepatocytes and restrict delivery to the marrow. This adds an anemia-of-inflammation component to ineffective erythropoiesis (15–17).

Splenectomy may further accentuate inflammation and thrombosis in thalassemia. Post-splenectomy patients often have higher IL-6 and TNF-α levels, reactive thrombocytosis and hypercoagulability. IL-6 promotes synthesis of fibrinogen and C-reactive protein, induces endothelial activation and upregulates tissue factor, creating a prothrombotic milieu. It may also contribute to immune dysregulation, alloimmunization and autoantibody formation.

### 4.3 Paroxysmal nocturnal hemoglobinuria

Paroxysmal nocturnal hemoglobinuria (PNH) is an acquired clonal hematopoietic stem-cell disorder caused by somatic PIGA mutations, leading to deficiency of glycosylphosphatidylinositol-anchored complement regulators and complement-mediated intravascular hemolysis (41). PNH is also associated with systemic inflammation and a high risk of thrombosis.

Complement activation in PNH triggers both hemolysis and coagulation. Thrombin generated downstream of complement activation induces IL-6 production by monocytes, macrophages and endothelial cells. IL-6 can in turn upregulate tissue factor and enhance thrombin generation, creating a positive feedback loop between complement, coagulation and inflammation. IL-6 also promotes hepatic synthesis of fibrinogen and other acute-phase proteins and contributes to endothelial activation and vascular permeability (42, 43).

Clinical studies have reported higher IL-6 levels in PNH than in healthy controls, with correlations between IL-6 and coagulation markers such as D-dimer and thrombin-antithrombin complexes (42, 43). Eculizumab reduces hemolysis, inflammatory stimulation and coagulation activation, and may indirectly lower IL-6. Newer proximal complement inhibitors, including iptacopan and danicopan, also attenuate hemolysis and thrombotic risk, although longitudinal IL-6 data are limited (44–47).

The clinical evidence for complement inhibition should also be interpreted in an evidence-based context. A Cochrane systematic review of eculizumab for PNH concluded that eculizumab improved quality of life, fatigue and transfusion independence, but that the evidence was insufficient to support or refute routine use because conclusions were based on a single small randomized controlled trial with important methodological limitations and low-to-moderate certainty of evidence (48). This review underscores an important distinction for the present article: reducing complement-mediated hemolysis is biologically and clinically plausible, but clinical effectiveness and certainty of evidence must be judged separately. It also highlights that any proposed additive role of IL-6 targeting in PNH remains unproven and would require prospective testing beyond optimized complement inhibition.

Genetic factors may modify IL-6 activity in PNH. IL-6 promoter polymorphisms, such as -174G/C, have been associated with IL-6 expression and PNH susceptibility in selected populations, although these findings require validation (49). Overall, IL-6 in PNH is best viewed as a secondary amplifier of complement-driven hemolysis and thrombosis. Whether IL-6-targeted or trans-signaling-selective approaches add benefit to optimal complement inhibition remains unknown.

### 4.4 Congenital red-cell membrane and enzyme defects

For several congenital hemolytic disorders, including red-cell membrane and enzyme defects, direct evidence linking IL-6 to disease progression or therapeutic response remains sparse. These conditions are therefore discussed primarily as examples of chronic hemolysis in which IL-6 may reflect secondary inflammatory signaling rather than a validated disease-modifying pathway. This distinction is important because persistent hemolysis can induce DAMP release, innate immune activation and hepcidin-mediated iron redistribution, but such associations do not by themselves establish IL-6 as a causal therapeutic target.

In congenital hemolytic anemias such as hereditary spherocytosis and other membrane or enzyme defects, persistent hemolysis can produce chronic low-grade inflammation with elevated IL-6 (50, 51). IL-6 may suppress erythroid progenitors, impair erythropoietin production and induce hepcidin, thereby worsening anemia beyond the primary red-cell defect. Observational data also suggest that higher IL-6 may be associated with allo-immunization or autoimmunization, although evidence remains limited (50).

Data on IL-6 in glucose-6-phosphate dehydrogenase deficiency are sparse and inconsistent. Some studies suggest altered IL-6 responses to infection, whereas others show largely preserved cytokine production (52). Current evidence is insufficient to support conclusions about IL-6-targeted therapy in this setting.

### 4.5 Hyperhemolysis syndrome

Hyperhemolysis syndrome is a rare but life-threatening transfusion complication, particularly in SCD, characterized by destruction of both transfused and autologous RBCs (53–55). Its pathogenesis is incompletely understood, but excessive macrophage activation in a cytokine-rich environment appears central. IL-6 may promote macrophage activation and phagocytosis, contributing to indiscriminate RBC clearance and abrupt hemoglobin decline.

Successful rescue treatment with tocilizumab in refractory cases supports IL-6 as a potential mediator, although evidence remains limited to case reports (56–58). This therapeutic signal is biologically plausible because hyperhemolysis may resemble an acute macrophage-activation phenotype rather than simple alloantibody-mediated hemolysis. Nevertheless, the rarity of the syndrome, concurrent use of corticosteroids, intravenous immunoglobulin, complement inhibition or other immunomodulatory treatments, and lack of prospective studies make it difficult to define the independent contribution of IL-6 blockade. At present, IL-6 receptor blockade should be considered a potential rescue approach only in carefully selected refractory cases.

Disease-specific roles of IL-6 in HAs were summarized in **Table 1**.

**Table 1:** Disease-specific roles of IL-6 in hemolytic anemias.

Disease context	Primary pathogenic trigger	Dominant IL-6 position	Main IL-6-linked pathway	Clinical implications
Warm AIHA	Loss of immune tolerance and IgG autoantibody production (1, 2)	Upstream immune amplifier	B-cell survival, plasma-cell differentiation, Tfh/Th17 activation and impaired Treg function (18–21)	IL-6 may correlate with disease activity and steroid resistance; IL-6 blockade remains investigational in primary wAIHA (21–23)
Castleman disease-associated AIHA/Evans syndrome	IL-6-driven lymphoproliferation, plasmacytosis and systemic inflammation (25–27)	Primary pathogenic driver	Hypergammaglobulinemia, autoantibody production and inflammatory cytopenias (25, 27)	Strongest rationale for IL-6/IL-6R blockade among hemolytic settings (28–31)
Sickle cell disease	Chronic hemolysis, free heme, vaso-occlusion and ischemia-reperfusion injury (3, 32)	Secondary inflammatory amplifier	TLR4 activation, endothelial activation, leukocyte recruitment and organ inflammation (6, 7, 33)	Potential target in selected complications such as acute chest syndrome or hyperhemolysis, but evidence remains low level (34, 35, 56)
Thalassemia	Ineffective erythropoiesis, transfusion exposure and iron overload (4)	Inflammatory modifier of erythropoiesis and iron handling	Hepcidin modulation, macrophage iron retention, endothelial activation and thrombosis (15–17, 38)	IL-6 may contribute to functional iron restriction and prothrombotic inflammation; targeting would likely require combination with disease-specific therapy (38–40)
PNH	Complement-mediated intravascular hemolysis and thrombin generation (41)	Thrombo-inflammatory amplifier	Complement-coagulation-inflammation feedback, endothelial activation and acute-phase response (42, 43)	Complement inhibition remains central; additive value of IL-6 targeting is unproven (44–47)
Congenital membrane/enzyme defects	Persistent low-grade hemolysis due to intrinsic RBC defects (50, 51)	Chronic inflammatory amplifier	Hepcidin induction, erythroid suppression and possible immune sensitization (50)	Evidence is limited; IL-6 may help explain inflammation beyond mechanical hemolysis (50, 51)
Hyperhemolysis syndrome	Transfusion, alloimmunity and macrophage activation (53–55)	Acute mediator of macrophage-driven RBC clearance	Cytokine-rich macrophage activation and phagocytosis of autologous and transfused RBCs (53–55)	Tocilizumab has shown rescue responses in refractory cases, but data are restricted to case reports and small series (56–58)

AIHA, autoimmune hemolytic anemia; iMCD, idiopathic multicentric Castleman disease; IL-6, interleukin-6; PNH, paroxysmal nocturnal hemoglobinuria; RBC, red blood cell; Tfh, T follicular helper cell; Th17, T helper 17 cell; Treg, regulatory T cell; wAIHA, warm autoimmune hemolytic anemia.

## 5 Therapeutic targeting of IL-6 and downstream pathways

Therapeutic implications of IL-6 biology in hemolytic anemias should be interpreted with caution. With the exception of IL-6-driven lymphoproliferative disorders such as Castleman disease, direct IL-6 or IL-6 receptor blockade remains investigational in most hemolytic settings. Current evidence consists largely of mechanistic studies, observational associations, and case reports or small series, particularly in refractory hyperhemolysis syndrome and immune cytopenias associated with systemic inflammatory disease. Therefore, biological plausibility should be clearly distinguished from demonstrated clinical effectiveness. Elevated IL-6 may identify inflammatory burden, but it does not by itself prove that IL-6 is causal or that IL-6 blockade will improve clinical outcomes. Accordingly, IL-6 should not be regarded as a validated universal therapeutic target across hemolytic anemias. Rather, its clinical relevance depends on whether it occupies a disease-driving, immune-amplifying, thrombo-inflammatory or macrophage-activation position in a given patient or disease context.

Future therapeutic development should therefore avoid an unselected strategy based solely on elevated circulating IL-6 levels and instead prioritize biomarker-defined populations. Potential enrichment markers include serum IL-6, CRP, soluble IL-6 receptor, soluble gp130, hepcidin, ferritin, transferrin saturation, plasmablast expansion, Tfh/Th17 signatures, macrophage activation markers, D-dimer, complement activation products and endothelial activation markers. Such stratification would help distinguish patients in whom IL-6 is likely to be a causal mediator from those in whom it is mainly an epiphenomenon of hemolysis or tissue injury (**Table 2****).**

**Table 2:** IL-6-directed and downstream therapeutic strategies in hemolytic anemias.

Strategy	Target or mechanism	Potentially relevant hemolytic setting	Current evidence	Key limitations and safety concerns
Tocilizumab	Blocks soluble and membrane-bound IL-6 receptor (10, 59)	MCD-associated AIHA, Evans syndrome, refractory hyperhemolysis syndrome and selected systemic autoimmune cytopenias	Case reports and small series suggest rapid hematologic improvement in selected refractory cases (28, 29, 31, 56–58)	No randomized trials in HA; infection risk, neutropenia, liver enzyme elevation and lipid abnormalities; responses often confounded by concurrent therapy (10, 59)
Siltuximab	Directly neutralizes IL-6 (10, 27)	Idiopathic MCD with AIHA or Evans syndrome	Case reports and real-world iMCD data support benefit in IL-6-driven disease (30, 60, 61)	Limited hemolysis-specific data; optimal duration and combination strategy remain uncertain (30, 60)
sgp130Fc/olamkicept-like approaches	Selectively inhibits IL-6 trans-signaling (13, 14)	Theoretical use in SCD, thalassemia or PNH with vascular inflammation	No direct clinical data in hemolytic anemias; rationale derives from IL-6 trans-signaling biology (11–14)	Requires preclinical validation; unclear effect on anemia, thrombosis and organ outcomes
JAK inhibitors	Inhibit downstream JAK/STAT signaling shared by IL-6 and other cytokines (11, 63)	Monogenic immune dysregulation with AIHA/Evans syndrome; selected refractory cytokine-driven cytopenias	Case-based evidence in STAT1/STAT3 gain-of-function disease and interferonopathies (63–66)	Broader immunosuppression; infection, thrombosis and potential malignancy risks; not IL-6-specific
Disease-specific therapy with indirect IL-6 modulation	Reduces upstream hemolysis, complement activation or inflammatory burden	Hydroxyurea or supportive therapy in SCD, iron-directed therapy in thalassemia, complement inhibitors in PNH	Stronger disease-specific evidence than direct IL-6 blockade in many non-autoimmune HA (32, 45–47) For eculizumab in PNH, a Cochrane review identified improvements in fatigue, quality of life and transfusion independence but emphasized that conclusions were limited by one small randomized trial and low-to-moderate certainty evidence (48)	May not fully suppress residual IL-6-driven inflammation; biomarkers are needed to identify persistent cytokine activity (33, 42, 43)

AIHA, autoimmune hemolytic anemia; HHS, hyperhemolysis syndrome; IL-6, interleukin-6; JAK, Janus kinase; MCD, multicentric Castleman disease; PNH, paroxysmal nocturnal hemoglobinuria; SCD, sickle cell disease; sgp130Fc, soluble glycoprotein 130 Fc fusion protein; STAT, signal transducer and activator of transcription.

### 5.1 Direct IL-6 or IL-6 receptor blockade

Targeting IL-6 has become established in several autoimmune and inflammatory diseases (10, 59). Tocilizumab blocks soluble and membrane-bound IL-6 receptor, whereas siltuximab directly neutralizes IL-6. Both provide proof of principle for IL-6-directed therapy in hemolytic disorders, but current evidence is mostly off-label and based on case reports or small series. Overall, the quality of evidence remains low, and conclusions should be considered preliminary.

#### 5.1.1 Tocilizumab

In AIHA, tocilizumab has mainly been used in patients with disease secondary to systemic autoimmune or lymphoproliferative disorders refractory to conventional therapy. In MCD-associated AIHA, tocilizumab has induced hematologic remission within weeks, with reductions in autoantibody titers and plasmablast counts (28, 29, 31).

Tocilizumab has also emerged as a potential rescue therapy for hyperhemolysis syndrome, particularly in SCD. Standard treatment with high-dose corticosteroids and intravenous immunoglobulin is not always effective, and further transfusion may worsen hemolysis. Several reports describe rapid cessation of hemolysis, stabilization or improvement of hemoglobin and resolution of inflammation after one or two doses of tocilizumab, including cases refractory to steroids, intravenous immunoglobulin and eculizumab (56–58).

Despite these encouraging observations, no randomized trials have evaluated tocilizumab in AIHA or hyperhemolysis syndrome. The reported patient numbers are small, and concurrent therapies often confound attribution of response. IL-6 blockade also carries risks of serious infection, neutropenia, liver enzyme elevation and lipid abnormalities. Tocilizumab is therefore most rational in settings where IL-6 appears to be an upstream driver of autoimmunity, such as MCD-associated AIHA or Evans syndrome, or where IL-6-dependent macrophage activation predominates, as in selected cases of hyperhemolysis.

#### 5.1.2 Siltuximab

Siltuximab directly neutralizes IL-6 and is approved for idiopathic MCD. Its use in hemolytic disorders has mainly been reported in MCD-associated AIHA and Evans syndrome. In refractory mixed AIHA associated with MCD, siltuximab has produced rapid and durable hemoglobin recovery after failure of corticosteroids, rituximab, intravenous immunoglobulin, erythropoietin and cyclosporine (30). Real-world and long-term data in idiopathic MCD further support sustained IL-6 neutralization as an effective strategy in selected IL-6-driven disease (60, 61).

These cases suggest that IL-6 neutralization can be effective when cytopenias are embedded in an IL-6-driven lymphoproliferative disorder. However, the number of reported cases remains small, and optimal dosing, duration and combination strategies are unknown. Comparative data between IL-6 neutralization and IL-6 receptor blockade in hemolytic disorders are lacking. In practice, agent selection is currently guided more by regulatory approval, availability, prior exposure and safety considerations than by direct comparative evidence.

#### 5.1.3 Trans-signaling-selective approaches

Because IL-6 trans-signaling is implicated in endothelial activation, leukocyte recruitment and chronic vascular inflammation, selective inhibition using soluble gp130-Fc fusion proteins, such as olamkicept, is an attractive strategy (14). These agents have not been tested in hemolytic anemias. In theory, they could attenuate vascular inflammation, thrombosis and organ injury in SCD, thalassemia or PNH while preserving some protective functions of classic IL-6 signaling. This approach is most appealing where IL-6 acts as a secondary amplifier of vascular injury rather than a primary driver of autoimmunity.

### 5.2 JAK/STAT inhibition

JAK inhibitors indirectly modulate IL-6 signaling by blocking downstream JAK/STAT activation shared by multiple cytokine receptors. Ruxolitinib and tofacitinib have been used in myeloproliferative neoplasms, autoimmune disease and monogenic immune dysregulation syndromes with hyperactive JAK/STAT signaling.

In β-thalassemia, ruxolitinib has been explored to reduce stress erythropoiesis and ineffective hematopoiesis. By inhibiting JAK2 downstream of the erythropoietin receptor, it may reduce expansion of immature erythroid progenitors and improve splenomegaly (62). *In vitro* data suggest possible induction of fetal hemoglobin, but clinical evidence remains limited and not specifically focused on IL-6 modulation.

More relevant to immune cytopenias, ruxolitinib has been used in severe AIHA or Evans syndrome associated with STAT1 or STAT3 gain-of-function mutations and other interferonopathies. In these disorders, JAK inhibition can suppress interferon- and IL-6-related pathways, normalize T-cell abnormalities and reduce autoimmunity (63–67). At present, JAK inhibitors are best considered for rare monogenic or highly refractory immune cytopenias in which IL-6 is part of a broader cytokine network. Their risks, including serious infection, opportunistic infection, thrombosis and potential malignancy signals, require careful patient selection and long-term monitoring.

### 5.3 Safety and limitations

Therapeutic targeting of IL-6 or JAK/STAT pathways in hemolytic anemias remains exploratory. Most published evidence consists of case reports and small uncontrolled series, with likely publication bias and heterogeneous outcome measures. Many patients received concurrent corticosteroids, rituximab, complement inhibition or other immunosuppressants, making it difficult to attribute responses to IL-6 blockade alone. Except for complement inhibition in PNH, where randomized evidence has been systematically reviewed but remains limited by the small size and methodological constraints of early trials, randomized controlled evidence for IL-6-directed therapy in hemolytic anemias is essentially absent. These limitations require cautious interpretation of apparent therapeutic responses.

Safety is a major concern. IL-6 blockade may impair host defense and mask typical inflammatory signs, delaying infection diagnosis. JAK inhibitors have recognized risks of serious infection, thromboembolic events and malignancy in some populations. Patients with hemolytic anemias may already have iron overload, organ dysfunction, immune suppression or vascular risk, potentially amplifying these hazards.

Future studies should use mechanistic enrichment and standardized endpoints. Stratification should consider whether IL-6 activity is predominantly autoimmune, hepcidin-mediated or thrombo-inflammatory. Baseline IL-6, sIL-6R, hepcidin, C-reactive protein, cytokine signatures and cellular immune profiles may help identify patients most likely to benefit. Clinically relevant endpoints should include hemoglobin response, transfusion requirement, hemolysis markers, thrombotic events, infection rates and durability of remission.

## 6 Conclusions and future directions

IL-6 provides a useful integrative framework for understanding the intersection between hemolysis, innate immune activation, iron restriction, adaptive immune dysregulation and thrombo-inflammation. However, its role is highly context dependent. In Castleman disease-associated autoimmune cytopenias, IL-6 may act as a primary pathogenic driver and is supported by the strongest therapeutic rationale. In primary AIHA, IL-6 is better positioned as an immune amplifier that may sustain autoreactive B-cell and T-cell responses in selected inflammatory phenotypes. In sickle cell disease, thalassemia and PNH, IL-6 currently appears more likely to function as a secondary inflammatory or thrombo-inflammatory amplifier than as an established treatment target. In hyperhemolysis syndrome, case-based experience suggests that IL-6 receptor blockade may be useful as rescue therapy in carefully selected refractory cases, but prospective validation is lacking.

Key unanswered questions remain. First, it is unclear whether IL-6 is a causal therapeutic target, a disease modifier or mainly a biomarker of inflammatory burden in each hemolytic disorder. Second, the biomarkers that best identify IL-6-dependent disease such as IL-6, CRP, hepcidin, ferritin require validation. Third, future studies should determine whether IL-6 blockade adds benefit to established disease-specific therapies, including immunosuppression in AIHA, transfusion and iron-directed strategies in thalassemia, hydroxyurea or anti-sickling therapy in SCD, and complement inhibition in PNH. Finally, prospective studies should include standardized endpoints for hemoglobin response, transfusion burden, hemolysis markers, thrombosis, organ injury, infection and durability of remission.

The next stage of the field should move from descriptive cytokine associations toward biomarker-stratified mechanistic and clinical studies. Such studies should define whether IL-6 elevation identifies a causal pathway, a compensatory inflammatory response or a marker of disease severity. Until these distinctions are clarified, IL-6-directed therapy outside established IL-6-driven disorders should be considered investigational and reserved for selected refractory cases within multidisciplinary clinical decision-making or prospective study frameworks.
